# An Evidence-Based Walking Program in Oregon Communities: Step It Up! Survivors

**DOI:** 10.5888/pcd17.200231

**Published:** 2020-12-10

**Authors:** Cynthia K. Perry, Laura P. Campbell, Jessica Currier, Paige E. Farris, Elizabeth S. Wenzel, Mary E. Medysky, Adrienne Zell, Miriam McDonell, Jackilen Shannon, Kerri Winters-Stone

**Affiliations:** 1 School of Nursing, Oregon Health & Science University, Portland, Oregon; 2 Oregon Clinical and Translational Research Institute, Oregon Health & Science University, Bend, Oregon; 3 North Central Public Health District–Public Health, The Dalles, Oregon; 4 Oregon Health & Science University–Portland State University School of Public Health, Portland, Oregon

## Abstract

Physical activity can help mitigate the long-term symptoms and side effects of cancer and its treatment, but most cancer survivors are not active enough to achieve these benefits. An evidence-based strategy to promote physical activity among adults is a community group–based walking program. However, many evidence-based programs do not achieve intended population health outcomes because of the challenges of real-world implementation. We used the Interactive Systems Framework for Dissemination and Implementation to conceptualize implementation of a capacity-building intervention to support delivery of a community group–based walking program. We adapted an evidence-based guide for community group–based walking programs for cancer survivors and their support network. We provided a capacity-building intervention (technical assistance and small-grant funding) and evaluated this implementation intervention. We assessed effectiveness of the intervention by measuring adoption, acceptability, appropriateness, feasibility, fidelity, implementation costs, and penetration through monthly progress reports, site visit observations, interviews, and a final report. Eight organizations received a small grant and technical assistance and implemented Step It Up! Survivors (SIUS). SIUS helped cancer survivors increase their physical activity, establish social connections, and be part of a supportive environment. Despite receiving monthly technical assistance, some grantees experienced challenges in recruiting participants, developing community partnerships, and adhering to the prescribed implementation plan. Implementation facilitators included community partners and specific components (eg, incentives for participants, webinars). Organizations needed different amounts and types of assistance with adaptation and implementation. Overall fidelity to SIUS ranged from 64% to 88%. Some integrated SIUS within existing organizational programming for sustainability. The provision of funding and technical assistance was a successful implementation intervention. Our results suggest a need to better tailor technical assistance while organizations are in the process of adapting, implementing, and sustaining an evidence-based program in their local communities.

**Summary**

**What is already known on this topic?**

Providing technical assistance and small-grant funding to community organizations is a promising approach to support implementation of evidence-based programs.

**What is added by this report?**

Community organizations are challenged by some aspects of implementing an evidence-based program, particularly balancing fidelity and adaptation and conducting a rigorous evaluation.

**What are the implications for public health practice?**

Difficulties experienced by some organizations in balancing fidelity and adaptation of the Step It Up! Survivors program indicate that flexible, individualized guidance and enhanced technical assistance are needed when they are in the process of adapting, implementing, and sustaining an evidence-based program locally.

## Introduction

In 2019, approximately 16.9 million cancer survivors were living in the United States, and their number is estimated to increase to 22.1 million by 2030 (1). Cancer survivors experience long-term symptoms and side effects of cancer and its treatment, including pain, fatigue, sleep disturbances, mood disturbances, reduction in quality of life (2), decreased physical functioning, and bone and muscle loss (3). Physical activity can mitigate many of these long-term symptoms and side effects (1,3,4). Additionally, regular sufficient physical activity may reduce the risk of recurrence, cancer mortality and all-cause mortality (1,3,4). The American Cancer Society recommends that cancer survivors engage in 150 minutes per week of moderate activity, such as brisk walking (4). The American College of Sports Medicine indicates 90 minutes of moderate level activity mitigates symptoms and side effects of cancer and its treatment (5). Less than half of cancer survivors are sufficiently physically active (6).

Group exercise is a promising approach for cancer survivors. Group and/or supervised exercise has resulted in greater improvements in primary outcomes (eg, fitness, muscle strength) compared with unsupervised and/or home-based interventions among cancer survivors (7). Group walking programs, an evidence-based strategy recommend by The Guide to Community Preventive Services (8), is a promising approach to enhance physical activity among cancer survivors because it is simple, geographically convenient, inexpensive, and suitable for most people. Community-based group walking programs enhance adherence because of the social support and cohesion developed among group members (9).

Public health programs, such as group-based walking programs, should reflect the best available evidence. However, many evidence-based programs (EBPs) do not achieve intended health outcomes because of challenges in implementation. Capacity-building interventions enhance community public health practitioners’ adoption and implementation of EBPs (10). The Interactive Systems Framework for Dissemination and Implementation (ISF) describes a process for translating research evidence and supporting community organizations to implement programs to achieve the intended outcomes with capacity building as the central component (11,12). The ISF describes 3 interacting systems: the Prevention Synthesis and Translation system, which distills research, the Prevention Support System, which provides capacity building, and the Prevention Delivery System, which delivers the program (12).

Standard approaches are needed to assess the implementation of evidence-based cancer prevention, control, and treatment interventions. Proctor and colleagues developed a taxonomy of implementation outcomes that includes acceptability, adoption, appropriateness, feasibility, fidelity, cost, penetration and sustainability (13). Using a standard approach to assessing the effectiveness of implementation of capacity-building interventions can allow comparisons across interventions and provide information on the best approaches to promote implementation of EBPs.

## Purpose and Objectives

The goals of this study were to 1) adapt an evidence-based guide for community walking programs for cancer survivors and their friends and family, 2) provide capacity-building support (technical assistance and small-grant funding) for community organizations to implement this program, and 3) evaluate the success of the implementation of the intervention.

We used the ISF to conceptualize implementation of a capacity-building intervention to support delivery of a community group–based walking program. We adapted an evidence-based guide for delivering group-based walking programs for cancer survivors and their friends and family and called the program Step It Up! Survivors (SIUS). We provided capacity-building support, technical assistance, and small-grant funding to community and public health organizations in Oregon.

We used a mixed-methods design to assess the implementation outcomes. We collected qualitative and quantitative data from several sources during the study period. The study was approved by the Oregon Health & Science University (OHSU) Institutional Review Board. The study, including planning, took place from July 2017 through February 2019.

## Intervention Approach

We completed implementation strategies and activities in each of the 3 interacting systems delineated in the ISF: The Prevention Synthesis and Translation System and the Prevention Support System were represented by OHSU and the Knight Cancer Institute. The Prevention Delivery System was represented by the community-based organizations.

### Prevention Synthesis and Translation System

**Figure 1:**
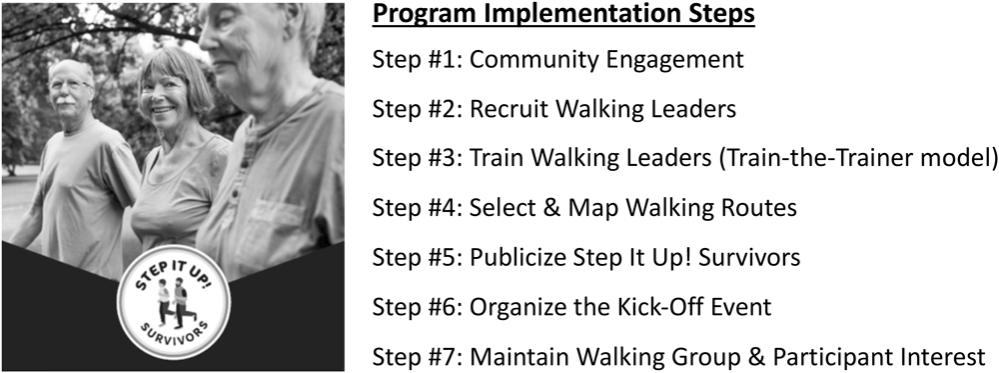
Cover of toolkit developed for Step It Up! Survivors walking program, Oregon, 2017–2019.

We identified the core elements of a walking program and its key characteristics to meet the needs of cancer survivors through a literature review. We searched PubMed by using the following search terms without limiting study year or study type: “group-based,” “community,” and “walking program.” We adapted an action guide developed by the Partnership for Prevention with support from the Centers for Disease Control and Prevention, *Establishing a Group Based Walking Program to Increase Physical Activity Among Youth and Adults* (14), to create a user-friendly toolkit for use by community-based organizations with programs designed for cancer survivors. Our toolkit (Figure 1) outlined 7 steps to implementing the program. It included a week-by-week timeline and guidance for completing each step and additional resources and deliverables for grantees. The program included friends and family members of survivors because of the importance of social support for behavior change.

### Prevention Support System

#### Innovation-specific capacity building

**Small grant funding.** We added a special request for applications to the OHSU Knight Cancer Institute Community Partnership Program (www.ohsu.edu/knight-cancer-institute/community-partnership-program-grants), which provides small grants ($10,000–$50,000) to community organizations. We issued a request for proposals for implementation of SIUS. Community groups, health and medical clinics, public health departments, and health systems in Oregon that did not have an active walking program were eligible to submit an application for a 1-year $15,000 grant, plus technical assistance. Before the application submission date, we hosted a webinar about the competitive funding process, and funding decisions were based on proposal review by members of our Cancer Prevention and Control Research Network’s Tribal and Rural Advisory Board, a previous Community Partnership Program grantee, and OHSU researchers. Final funding decisions were made by Community Partnership Program leadership. Funding was provided from January 2018 through December 2018.

**Technical assistance.** The team of researchers worked with grantees to implement the SIUS, including assistance with any relevant regulatory approvals. In March 2018, representatives from each organization attended a train-the-trainer webinar on how to train local community members to be walking group leaders. We facilitated 8 monthly, 60-minute webinars on recruitment, retention, and motivation of walking group leaders and participants, adaptation logs, and program sustainability. Each month, a different grantee made a 30-minute presentation on various topics (eg, data collection techniques, recruiting and retaining walking participants, engaging community partners), leaving 30 minutes for discussion facilitated by the research team. The webinars provided an opportunity for organizational leaders to learn from and support each other. Additionally, we responded to questions and provided individual guidance to grantees via telephone calls and email.

#### General capacity building

In January 2018, we held a training session developed by the Cancer Prevention and Control Research Network, Putting Public Health Evidence into Action (www.cpcrn.org/training). This 1-day in-person training session provides instruction on implementation of EBPs in public health. This instruction was applied by grantees to the SIUS program, but the skills could also be applied by grantees in future programs.

### Prevention Delivery System

Each grantee was responsible for implementing the SIUS in their community using the toolkit. Program Implementation was divided into 4 phases. The first 3 months involved start-up and program planning, which encompassed the first 6 of 7 steps: engaging the community; recruiting walking leaders; attending a train-the-trainer session; training the walking leaders; selecting and mapping walking routes, securing indoor location for use as needed; publicizing SIUS; and organizing a kick-off event. The second 3 months involved implementation of the program by holding weekly community walking groups for cancer survivors, their family and friends (step 7), continuing to publicize and recruit participants, maintaining interest and attendance in the program, and continuing to engage with the community. The third phase was a 2-month maintenance phase, and the final phase was 2 months for follow-up. In addition, each organization had 2 months to complete a final report.

## Evaluation Methods

We assessed effectiveness of the implementation intervention by measuring acceptability, adoption, appropriateness, feasibility, fidelity, implementation costs, penetration, and sustainability as described by Proctor and colleagues (13), through monthly progress reports, site visit observations, interviews, and a final report (Table 1).

**Table 1:** Implementation Outcome Definitions and Data Sources for Implementation of an Evidence-Based Walking Program in Oregon Communities: Step It Up! Survivors, 2018

Outcome	Definition	Data Sources
Acceptability	Perception among stakeholders that the program was agreeable and satisfactory	Final project report; satisfaction survey; attendance records
Adoption	Uptake of the program by the organization and community	Grant application; monthly progress report
Appropriateness	Perceived fit, relevance, and compatibility of the program for a given setting	Final project report; monthly progress report; satisfaction survey
Feasibility	The extent to which the program can be carried out	Monthly progress report; final project report
Fidelity	The degree to which the program was implemented as prescribed	Site visits; interviews; adaptation logs
Implementation costs	The costs of implementing the program	Monthly progress report; final project reports
Penetration	The degree to which the program is integrated within the setting	Final project report; monthly progress report
Sustainability	The extent to which the newly implemented program will be maintained or institutionalized	Final project report

**Community organization reports.** Monthly grantee progress reports described strategies for publicizing SIUS, tactics and incentives used to motivate participants and leaders, walking route, number of groups held, and attendance. A final report gathered data on SIUS objectives and outcomes (eg, create a safe and supportive environment for cancer patients, survivors, and their friends and family to come together for socializing through movement), project reach (eg, number of unique individuals attending groups), community partnerships (eg, number and role of community partners), strengths and benefits of the project, barriers, challenges and lessons learned, future plans, and evaluation of the technical assistance (eg, webinars, trainings, toolkit). The monthly and final reports were completed in a secure online database. Additionally, the grant applications provided information on the grantee (eg, size, rural/urban).

**Fidelity assessment: observation and stakeholder interviews.** For each grantee, research staff members observed a walking group and interviewed the program manager to assess fidelity. During the observation, the researchers completed a fidelity checklist of the key components of conducting a walking group outlined in the toolkit. Key components included characteristics of the walking leaders, the routes, use of team building and social support strategies, and accessibility and aesthetics of walking routes.

One research staff member conducted 30- to 60-minute semistructured interviews with key leadership at each organization and a second staff member completed the fidelity checklist and took notes during the interview. Key components of implementation described in the toolkit that were not possible to observe during the walking group were asked about during interviews, including types of stakeholders engaged in planning and implementation, identification and training of walking group leaders, alternate routes for inclement weather, minimizing loss of interest, and resources used to plan routes.

**Adaptation logs.** We provided organizations with an adaptation log to record adaptations to the program, the date and description of the adaptation, reason for the adaptation, and its level of importance. These logs allowed for comparison across organizations.

**Walking group participant survey.** Walking group leaders invited cancer-survivor participants to complete an online survey at baseline, 3 months, and 6 months. The survey included 11 statements about their experience and satisfaction with SIUS; respondents rated agreement on a scale from 1 to 6 (1 = strongly disagree, 6 = strongly agree). At baseline, the survey also collected information on cancer-survivor participants’ demographic characteristics (age, race, ethnicity, highest academic degree attained, marital status, employment status), height and weight, and cancer (type of cancer and number of years since diagnosis).

### Data analysis

**Qualitative data analysis.** We exported notes taken during the leadership interviews and coded them line by line. We exported text from the final project reports into Dedoose version 8.1 (Dedoose, LLC), a web-based program for analyzing qualitative and mixed-methods data. Two researchers coded the texts independently and met to reach agreement. The coding focused on implementation outcomes, barriers, and facilitators.

**Quantitative data analysis.** We exported quantitative data from the monthly progress reports and site visits into R statistical software version 3.5.1 (R Foundation for Statistical Computing), data from the online participant surveys into Stata version X (StataCorp LLC), and data from the fidelity checklist and monthly and final reports into Excel (Microsoft). We calculated descriptive statistics for each organization.

Walking group attendance was recorded each month by each organization. The average weekly attendance is the mean number of people attending a walking session across all groups hosted by an organization. Some organizations had more than one walking group that met weekly. The average monthly attendance is the average total attendance for all organizations across weeks in a particular month.

We measured fidelity to the SIUS implementation toolkit by using a checklist, which we completed during site visits (observations of the group walks), and an interview guide, which we used during interviews of organizations’ leadership. We calculated a fidelity score for each of the 7 steps of program implementation as a percentage. The checklist included 9 yes/no questions and 12 questions answered on a scale of 1 to 5; the checklist was also used to record the location and date of the site visit. To assign questions equal weight among the fidelity scores, we coded the rating-scale questions from 1 to 5, and we coded dichotomous questions yes = 5 and no = 1. We did not count responses marked as not applicable or missing in the calculation of the fidelity percentage. This method is commonly used in analysis of survey data (15).

**Mixed-methods analysis.** We placed the coded text and the quantitative results into an implementation outcomes matrix for further analysis. The mixed-methods analysis for organizational-level data spanned 3 areas: measuring program success using attendance data for each organization, assessing fidelity to the SIUS toolkit, and analyzing strategies and tactics to recruit and incentivize walking leaders and participants. Walking group attendance was a quantitative measure of program success, with larger attendance numbers indicating better program engagement. A higher fidelity percentage indicated greater fidelity to the SIUS toolkit. Strategies to recruit and incentivize walking leaders and walking group participants were deduced from the monthly reports, and qualitative themes were deduced from site visit data and monthly reports. We created a case-based matrix with the organizations in rows and the strategies in columns.

## Results

### Cancer survivor survey results

Sixty cancer-survivor participants completed eligibility screening, 35 met eligibility requirements, 30 consented to participate and completed the baseline survey, and 17 completed the survey at 3 and 6 months. Eligibility requirements included a cancer diagnosis, participation in an SIUS walking group, being aged 18 or older, and ability to speak English. Grantees did not prioritize completion of the online surveys.

Of the 30 cancer-survivor participants who completed the baseline survey, the average age was 60.4 (range, 44–79). Most were non-Hispanic (n = 28) and White (n = 28), had a bachelor’s degree or higher (n = 20), and were married (n = 26). About half were retired (n = 13), and half were employed full-time or part-time (n = 12). The most common form of cancer was breast cancer (n = 21) with time from diagnosis ranging from 4 months to 30 years.

### Implementation outcomes

**Adoption.** Ten organizations responded to the request for proposals for implementing SIUS; 8 organizations received awards. Organizations that received awards were local health departments (n = 3), community cancer centers (n = 3), a nonprofit organization that supports cancer survivors (n = 1), and a physical therapy practice (n = 1); 6 were in rural communities.

**Figure 2:**
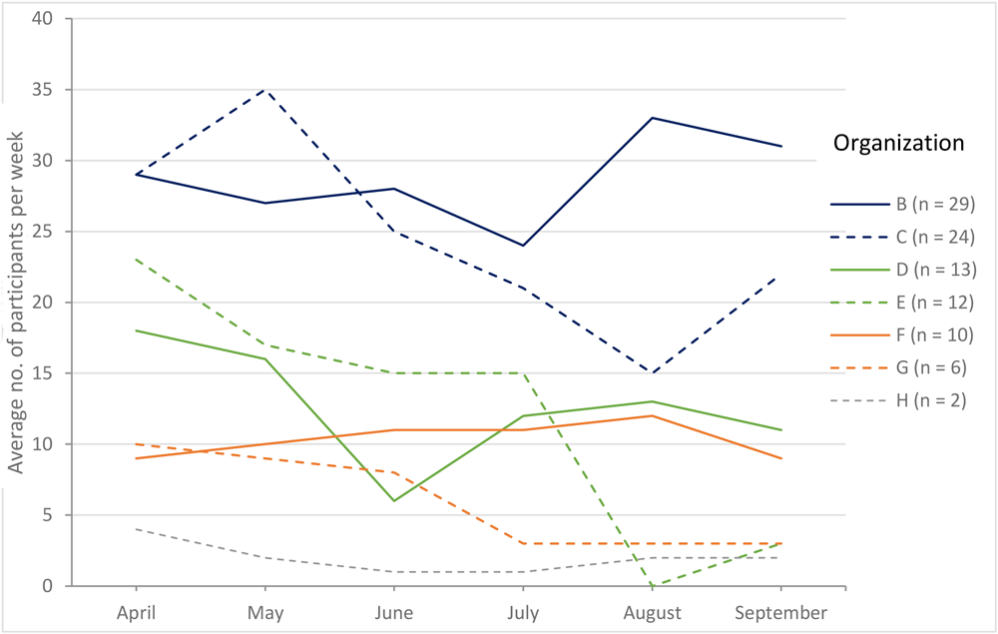
Average weekly attendance in walking group by organization and by month.

**Acceptability.** The average weekly attendance varied within and across organizations (Figure 2). One organization followed a different protocol to record walking group attendance than the other 7 organizations; we did not include these data in our analysis. The number of unique participants across all organizations was 258 and included cancer survivors, their family and friends, and community members. The median number of participants across all organizations was 34. The median number of monthly walking group attendees per organization ranged from 6 (Organization H) to 114 (Organization B). The median weekly attendees per group was 12 and the average weekly attendance per organization was 13.7.

**Table:** 

Organization	April	May	June	July	August	September
Organization B	29	27	28	24	33	31
Organization C	29	35	25	21	15	22
Organization D	18	16	6	12	13	11
Organization E	23	17	15	15	0	3
Organization F	9	10	11	11	12	9
Organization G	10	9	8	3	3	3
Organization H	4	2	1	1	2	2

Overall participant feedback was positive. Six organizations reported that their participants enjoyed the program and kept coming back. Additionally, organizations reported that walking group participants developed connections with each other. Anecdotally, cancer survivors reported having stronger social connections and a more positive outlook on life than before participating in SIUS and the value of receiving and giving peer support to their fellow cancer survivors. One community organization reported the following: “There was an overwhelming support from individuals volunteering to become walking leaders, as well as walking participants. The success led to friendships and socializing. There is a core group of walkers that faithfully attend and help welcome in new walkers.” Another stated, “The greatest success was the social connections created. Everyone agreed that being with and meeting others was a huge highlight of the program.”

Of the 17 participants who completed the online survey at 3 months, 16 agreed or strongly agreed that they were satisfied with the walking leader, the walking routes, and the program. At 6 months, all 17 survey respondents slightly agreed, agreed, or strongly agreed that they were satisfied with the walking leader, the walking routes, and the program.

**Appropriateness.** Walking was generally considered by organizations’ leadership and walking leaders an appropriate form of exercise for cancer survivors because they could move at “their own pace and ability.” Five of 8 sites reported that their participants experienced increased physical activity, health benefits, and social connectedness and engagement. As one organization stated, “Having this weekly walking group has created a safe space for our patients who are currently undergoing treatment to come and move at their own pace and ability, while meeting and talking with survivors who have been in their shoes.” Another stated, “Participants reported significant improvements to health and cited increased social engagement as their primary participation benefit.”

At 6 months, 15 of 17 survey respondents agreed that the program helped them increase their physical activity, 17 agreed the program was a good way to be physically active, and 16 reported feeling connected to other members of the walking group who were supportive of each other.

Five organizations stated that participants wanted the groups to continue after the end of the project. Reasons provided by the organizations for discontinuing the walking groups included climate, (ie, high temperatures and unsafe air quality from wildfires), lack of commitment from organizational leadership, challenging retention during the summer because of summer travel and vacations, and weak community partnerships.

**Feasibility.** All organizations reported that they implemented at least 1 walking group during the study period. Some organizations experienced low attendance and challenges with keeping walking leaders engaged over the 3 months (Table 2). Challenges in retaining walking leaders included life changes (eg, changing job, moving away), summer travel, and physical limitations (some walking leaders were receiving cancer treatment that affected their ability to fulfill their role). All organizations found the data reporting requirements of the grant burdensome.

**Table 2:** Challenges and Facilitators, by Implementation Step and by Organization, During Implementation of an Evidence-Based Walking Program in Oregon Communities: Step It Up! Survivors (SIUS), 2018

Organization: Rural/Urban and Type	Step 1: Community Engagement	Step 2: Recruit Walking Leaders	Step 3: Train Walking Leaders	Step 4: Select and Map Walking Routes	Step 5: Publicize SIUS	Step 6: Kick-off Event	Step 7: Maintain Walking Group and Participant Interest
**Organization A: Rural, nonprofit organization**							
Facilitator	Program promotion; garnered support of community leaders; established community partners	Committed walking group leaders	Walking group leader toolkit	Public partnership with community partners provided walking routes and maps	Promoted walking group among cancer survivor support groups	None reported	Solicited and integrated input from walking group leaders for quality improvement; integrated program into organization’s budget; support from organization’s leadership
Challenge	Community engagement required significant investment of staff time	None reported	None reported	None reported	None reported	None reported	None reported
**Organization B: Rural, nonprofit**							
Facilitators	Community partnerships resulted in exceeding expectations for walking group participation; Program built on and enhanced existing community partner walking and physical activity programs to create awareness and participation countywide	Community partner provided a volunteer staff member who helped recruit walking leaders and supported program coordination; Celebration of walking group leaders at a community cancer tribute event co-hosted by and the American Cancer Society revitalized partnership between the two organizations and renewed interest in community for programs for cancer survivors	Community partner helped recruit walking group leaders	Community partner provided walking group venue, mapped walking routes, registered participants, and reported attendance data from their group to project coordinator; Walking group leader toolkit	None reported	None reported	Participants liked walking venues/routes; Participants established important social bonds and were the impetus to sustain the program
Challenges	None reported	None reported	None reported	None reported	None reported	None reported	None reported
**Organization C: Urban, for-profit**							
Facilitators	Recruiting through existing cancer survivor support groups	Community of active cancer survivors ready and willing to lead walking groups	Walking group leader toolkit	Parks and Recreation Department active and supportive in developing walking group routes	Targeted recruitment to cancer survivors and their networks	None reported	Program organizer participated as active member in walking group; Participant incentives
Challenges	None reported	None reported	None reported	None reported	Flyers were not an effective recruitment tool	None reported	Low walking group participation; walking leader was in active cancer treatment limiting ability; Inability to continue providing participant incentives
**Organization D: Rural, public health/government**							
Facilitator	None reported	Recruited at community events and cancer center; Cancer center provided walking group leaders	Walking group leader toolkit	County Sherriff provided guidance in determining walking routes	Cancer center and community partners publicized SIUS	Offered flexibility by hosting 2 kick-off events	Community partner provided incentives to participants
Challenge	Finding community groups/organizations interested in partnering on this project	None reported	None reported	None reported	None reported	None reported	Difficulty finding time and location amenable to all participants
**Organization E: Rural, nonprofit**							
Facilitator	Interest in the community to participate; Participant incentives	Support from individuals volunteering to become walking leaders and participants	Walking group leader toolkit	Community partners located indoor walking route as alternative for inclement weather	Cancer center publicized SIUS	Kick-off event used to increase awareness and number of participants	Friendships, opportunity to socialize, and peer support from cancer survivors led to sustaining walking groups; Cancer Center absorbed advertising and participant incentive costs to sustain program
Facilitator	None reported	None reported	None reported	None reported	None reported	None reported	Hazardous air quality from wildfire smoke led to postponement of walking groups for a period of time
**Organization F: Rural, public health/government**							
Facilitator	Community nonprofit and for-profit partners promoted SIUS and supported in recruiting walking group participants and leaders	Adopted a flexible recruitment strategy through social media, in-person direct contact, and community partners	Walking group leader toolkit	None reported	Promoted SIUS through Facebook, local recreation activities guide, and presentations to service groups	Community partner donated food	Yoga studio and a local gym offered discounts for walking group participants; community parks and recreation department assumed program operations and management
Challenge	Lack of follow-through promoting SIUS among some community partners; Problematic to recruit cancer survivors because no cancer center or oncology practices are in the community	None reported	None reported	None reported	None reported	None reported	None reported
**Organization G: Rural, public health/government**							
Facilitator	Higher education institution was an important community partner	Community organizations helped recruit walking leaders	Walking group leader toolkit	Community partners provided walking routes on their property; Walking routes included indoor and outdoor options	Community partners promoted program internally and encouraged participation	Community partners donated gift cards for walking group participants	None reported
Challenge	None reported	Retaining walking group leaders was difficult	None reported	None reported	None reported	None reported	Walking group leader inability to continue in role led to group disbanding; Walking group attendance declined during summer months; Program suspended because of staff turnover and inability of other staff in organization to assume program management
**Organization H: Urban, nonprofit**							
Facilitator	Developed SIUS marketing materials and distributed to oncology centers in a metro area	Paid walking group leaders a stipend	Walking group leader toolkit; weekly informational sessions on fitness (eg, hydration, safety, shoes, nutrition)	Used “Map My Walk” app for walking route flexibility and variation	Created and distributed recruitment materials through various media sources (flyers, posters, social media, neighborhood group meetings); Welcome kits distributed to participants	None reported	Walking groups scheduled at 2 locations, with a selection of multiple days and times (weekday, weekend, morning, and evening)
Challenge	Some community groups unwilling to share information and allow access to survivor groups	None reported	None reported	None reported	None reported	None reported	Program ended prematurely because it lacked sustainable program management leadership

All organizations used the strategies suggested in the toolkit to publicize walking groups. The most common strategies were word of mouth, social media, and posters around the community. Other strategies for recruitment and retention included advertising with local media and engaging other community organizations or stakeholders. The grant required use of participant incentives to encourage retention; grantees used raffles, gift cards, and promotional products. One organization commented, “Program participation incentives were cited [by participants] as being an important element of the program’s success, making it fun to participate.”

Despite these strategies, organizations that were not connected with a cancer center reported difficulties reaching cancer survivors. Three organizations described challenges with recruitment. Five organizations expanded walking groups to include community members interested in prevention and making healthy choices. One organization reported challenges with recruiting walking leaders, and 1 organization reported challenges establishing community partners. All organizations used outdoor walking routes, and 4 organizations secured indoor locations for inclement weather (eg, poor air quality from forest fires, high ambient temperatures), allowing weekly groups to continue.

**Fidelity.** Fidelity was the degree to which the SIUS program was implemented as delineated in the toolkit. The number of adaptations across the 7 steps ranged from 0 to 4 (Table 3). Fidelity ranged from 62% (step 1; 225 of 360 possible points) to 86% (step 6; 212 of 245 possible points). Not counting missing responses and those marked not applicable toward scores, average fidelity across the steps for each organization ranged from 64% (163 of 255 possible points) to 90% (230 of 255 possible points). We asked grantees to propose and discuss adaptations with the research team to determine whether the proposed adaptation would affect the evidence-based components of the program; we planned to approve adaptations determined not to have an effect. This discussion often occurred, but the research team was not always aware of adaptations until after they had been made; some implemented adaptations were not approved by the research team.

**Table 3:** Step It Up! Survivor Adaptations, by Program Implementation Step, During Implementation of an Evidence-Based Walking Program in Oregon Communities: Step It Up! Survivors, 2018

Implementation Step	No. of Adaptations	Adaptation Description Reported by Organization
Publicize Step It Up! Survivors (Step 5)	4	Changes to layout, design, and content of promotional flyer
Maintain walking group and participant interest (Step 7)	3	Increased frequency of walking group meeting sessions, changed time of walking group meeting, implemented protocol for walker to maintain participation while on vacation
Train walking leaders (Step 3)	1	Trained new walking group leaders after program implementation began
Select and map routes (Step 4)	1	Added new walking routes after program implementation began
Organize the kick-off event (Step 6)	1	Liability language added to walker registration form
Community engagement (Step 1)	0	None
Recruit walking leaders (Step 2)	0	None

Five organizations implemented program adaptations. The most common adaptations were aimed at increasing reach, receptivity, and participation, including design changes to SIUS outreach materials, such as expanding audiences to include all community members interested in becoming more physically active and social and increasing the frequency of walking group meetings to twice per week per the interest of walking group participants. One adaptation fundamentally changed SIUS from a group-based to an individual-based walking program: the definition of attending a walking group was changed to include participants who sent an email to the walking group leader indicating that they had walked that week. Although this adaptation was viewed as more inclusive, the expanded definition conflicted with a key premise of SIUS about the importance of the group in providing social support, connectedness, and accountability. This adaptation was not pre-approved.

**Cost.** Each organization received approximately $15,000 to implement SIUS; this amount varied slightly depending on the budget requested by the organization. The grant required that at least $1,000 be allocated for participant incentives. Organizations also received in-kind donations from community partners (eg, space, personnel paid by a partner, volunteer hours). All organizations reported implementation of SIUS within the budget as planned, including in-kind donations.

**Penetration.** The number of walking groups held in each organization ranged from 1 to 9, with a mean of 5.2 walking groups per organization. One organization stated, “We set a goal of establishing 1 walking group and significantly exceeded our expectations with 5 active walking groups in our community.”

Two organizations reported incorporating the walking program into existing organizational structures. One organization reported that SIUS was co-promoted with another well-known wellness program in the organization, which contributed to successful recruitment. As one organization stated, “This walking group has become a part of the survivorship and cancer support/education group program that is led by the Oncology Social Worker, which promotes the likelihood of it continuing on into the next year.”

**Sustainability.** Four organizations reported that their program was highly likely or somewhat likely to be active 1 year after the funding period. These organizations reported that the largest contributing factors to expected sustainability were integration of the walking groups into existing programs (within their own organization or a community partner) and participant enjoyment. Another contributing factor was the use of volunteers to lead the walking groups. One organization noted, “[The city’s] recreation program has taken on the long-term coordination of Step It Up! Survivors in our town. The group is peer led and does not rely on paid staff [to lead the walking groups].”

## Implications for Public Health

This capacity-building intervention of providing technical assistance and small-grant funding was a successful approach to promoting implementation of an evidence-based community walking program for cancer survivors, their friends, and families. The 8 grantees varied in their capability to achieve success, suggesting the need for tailored technical assistance. Fidelity to the toolkit varied across the 7 steps in the program implementation and across the 8 organizations, suggesting the need for more clarity, more education about the importance of fidelity, and more individualized guidance on balancing fidelity and adaptation. Integration of SIUS into existing programs in the organization and participant enjoyment contributed to the likelihood of sustaining SIUS for at least 1 year beyond the funding period.

Six of the 8 organizations that implemented SIUS were in rural (Rural–Urban Continuum Codes 4–9) communities (16). Rural residents of the United States experience health disparities because of geographic isolation. Compared with their urban counterparts, they have lower socioeconomic status, fewer job opportunities, higher rates of health risk behaviors, limited access to health care specialists and subspecialists, and less likelihood to have employer-provided health insurance coverage; if they are experiencing poverty, they are often are not covered by Medicaid (17–19). Cancer survivors living in rural areas are diagnosed at later stages of disease and have more barriers to cancer prevention, control, and treatment than their urban counterparts (17,19). SIUS provided a supportive health promotion activity to cancer survivors, their family, and friends in an environment where health disparities persist.

Organizations commented that walking groups were an important source of social support for cancer survivors. Extension of the walking groups to cancer survivors’ friends and family and, in some locations, the broader community expanded the network of social support.

Capacity-building interventions providing technical assistance and/or small-grant funding have been found to enhance the adoption and implementation of EBPs (10,20–24). Similar to this study, other mini-grant programs have resulted in the recipient organizations building and strengthening partnerships with other community organizations (20,24). However, community organizations are typically set up for program delivery and not for evaluation; thus, it was challenging for community organizations receiving capacity-building interventions to complete the evaluation steps (eg, data collection, data analysis). All grantees reported that they did not prioritize participants completing the online surveys, as reflected in the low number who completed eligibility screening, and they found the grant reporting requirements burdensome. Similarly, in a mini-grant program, community organizations found it challenging to prioritize data collection for evaluation (23), and in a countywide mini-grant initiative, organizations did not have the capacity for evaluation (eg, lack of time, skill, and resources) (25). It is critical to determine the effectiveness of capacity-building interventions to enhance organizational capacity to adopt, implement, and sustain delivery of EBPs as well as ascertain the effectiveness of the EBP to achieve desired outcomes in the local setting. Thus, the Prevention Support System delivering the capacity-building intervention may need to provide additional support for data collection and evaluation to ensure evaluation of effectiveness.

Although key leadership from each grantee received training on fidelity and adaptation, we found a range of fidelity to and adaptations of the SIUS toolkit. A potentially inappropriate adaptation could compromise an underlying evidence-based component of a group-based walking program, as we found in the broadening of the definition of a walking group attendee. In a mini-grant program in which recipients received training on adaptation and fidelity of evidence-based interventions, the organizations made substantial alterations that might have changed the evidence-based components of the programs (22). In another, the grantees were challenged by balancing fidelity with adaptation, and some grantees dropped core program elements (26). Although retaining the core elements that produce the desired outcomes from the EBPs is critical, some adaptation is necessary to enhance a program’s relevance, community reach, alignment with local resources, and program sustainability (26). This balancing of fidelity and adaptation is difficult and requires careful consideration of the underlying theory and evidence base of the program as well as the community needs, desires, barriers, and facilitators to program implementation. The challenges of balancing fidelity and adaptation of EBPs, despite generalized training on these concepts, suggests that in addition to generalized training, individualized local guidance on fidelity and adaptation is needed. Our rural SIUS grantees faced several challenges, including a small number of community partners with whom to collaborate in program promotion and recruitment of walking leaders and participants. Additionally, Oregon had an active wildfire season in 2018. Poor air quality was more prevalent in rural locations than urban locations and affected scheduled walking sessions.

We found that community organizations with previous program implementation experience were able to build on that experience and move through the steps in the SIUS toolkit better than organizations with less experience. Although we delivered technical assistance through multiple modalities, including telephone calls, emails, training sessions, and monthly webinars, we did not tailor our approach to the experience of each organization. Providing technical assistance in a flexible manner that aligns with and addresses organizational realities (eg, leadership, resources) and is both relationship building and content driven has been effective in promoting implementation of EBPs (21,27). Assessing community and organizational readiness for implementing an EBP and tailoring technical assistance approach, type, and intensity to that level of readiness could enhance the effectiveness of the capacity-building intervention and harmonize the overall allocation of resources for the intervention.

Our study has several limitations. It was conducted in Oregon only, so results may not be generalizable to other areas of the United States. We did not audio record interviews with key stakeholders and despite careful notes taken during the interviews, some responses from interviewees may not have been fully reflected. Because of a short funding cycle, we could not follow up 1 year after the start of the walking groups to assess sustainability. The community organizations had challenges recruiting and retaining cancer survivors, and most opened the groups to any community member. Strengths of this study include use of theoretical framework, ISF (12), to conceptualize the capacity-building intervention; the implementation and evaluation of a community walking program for cancer survivors, a group for whom few community programs exist, particularly in rural areas; and the use of implementation outcomes suggested by Proctor and colleagues (13). Overall, we believe this study provides support for the use of capacity-building interventions to promote the implementation of EBPs by community organizations.

Use of the ISF helped us to conceptualize the elements and relationships of key stakeholders (eg, universities, community organizations) involved in providing a capacity-building intervention aimed at disseminating and implementing an evidence-based community health walking program for cancer survivors. The capacity-building intervention (technical assistance and small-grant funding) was a successful approach for dissemination and implementation of SIUS. Difficulties experienced by some organizations in balancing fidelity and adaptation of the SIUS program indicates that flexible, individualized guidance and enhanced technical assistance are needed while organizations are in the process of adapting, implementing, and sustaining an EBP locally. Use of capacity-building interventions to promote and guide the implementation of EBPs by community organizations is an effective method to achieve a program’s intended population health outcomes. Members of this research team have applied for funding to expand the scope of group walking programs for cancer survivors, their family and friends, as well as community members in rural locations throughout Oregon.
